# A combined designed CSP and Pfs48/45 infection and transmission blocking vaccine for malaria

**DOI:** 10.1038/s41541-025-01262-2

**Published:** 2025-09-02

**Authors:** Richi Gupta, Thayne H. Dickey, Nichole D. Salinas, Palak N. Patel, Rui Ma, Dashuang Shi, Myesha Singleton, Tarik Ouahes, Thao P. Pham, Kazutoyo Miura, Carole A. Long, Lynn E. Lambert, Niraj H. Tolia

**Affiliations:** 1 Laboratory of Malaria Immunology and Vaccinology, National Institute of Allergy and Infectious Diseases, National Institutes of Health, Bethesda, MD USA; 2 Laboratory of Malaria and Vector Research, National Institute of Allergy and Infectious Diseases, National Institutes of Health, Rockville, MD USA

**Keywords:** Protein vaccines, Protein vaccines, Malaria, Infection

## Abstract

The multiple stages of the malaria parasite life cycle hampers vaccine development. Combining a pre-erythrocytic antigen with a transmission-blocking antigen would target two independent stages of the life cycle for disease control, resulting in a multistage vaccine that can prevent infection and disease transmission simultaneously. Here, we generated a self-assembled ferritin nanoparticle vaccine that simultaneously presents designed immunogens CSPj5c and 17-4 from the infection-blocking circumsporozoite and the transmission-blocking Pfs48/45 antigens. These immunogens were designed, through structure-based approaches, to retain protective epitopes and confer protection upon vaccination. Immunization with CSPj5c-17-4-ferritin nanoparticles conferred protection against challenge with transgenic sporozoites expressing *Plasmodium falciparum* CSP in mice, and purified IgGs from immunized rabbits elicited potent transmission-reducing activity. Addition of the engineered 17-4 improved the immune responses to CSPj5c and protection from sporozoite challenge. CSPj5c-17-4-ferritin is therefore a promising multistage malaria vaccine with a potential role in malaria control.

## Introduction

Malaria is the leading cause of death among children, infants, and pregnant women in Africa with 249 million malaria cases worldwide and 608,000 malaria deaths in 2022^1,2^. Numerous innovative strategies for drug treatment, vaccine development, and intervention measures have been explored to prevent, control, and treat malaria. Although these strategies have reduced the mortality rate, the emergence of drug resistance confounds control efforts. Malaria vaccines will aid in ongoing efforts, but limitations remain due to the complex lifecycle of *Plasmodium* parasites and a poor understanding of immune response to malaria infection. In the human host, the *Plasmodium* parasite lifecycle consists of the pre-erythrocytic and erythrocytic stages. The pre-erythrocytic stage begins when an infected mosquito bites a human and injects sporozoites into the bloodstream. These sporozoites travel to the liver, invade hepatocytes, mature into schizonts and later rupture to release merozoites into bloodstream. During the erythrocytic stage, merozoites infect red blood cells (RBCs), multiply and release more merozoites in the blood resulting in clinical symptoms of malaria. Some merozoites circulating in the blood develop into gametocytes. Transmission occurs when gametocyte-containing RBCs are ingested by a mosquito through a bite followed by maturation into male and female gametes prior to the sporogonic cycle to produce sporozoites that migrate to salivary glands to infect another human. Human antibodies are also ingested by the mosquito and can target gametocytes in the mosquito midgut to block transmission. Development of a multistage vaccine that targets two or more lifecycle stages of malaria is a promising approach and an excellent tool to support malaria elimination. An effective multistage vaccine will disrupt the lifecycle and aid in reducing both disease burden and transmission.

The World Health Organization has recommended the use of malaria vaccines comprised of single stage antigens for the prevention of *P. falciparum* malaria in children living in moderate to high transmission regions^3^. RTS,S/AS01, also known as Mosquirix, and R21/MatrixM are licensed malaria vaccines recommended for use in children^4,5^. Both vaccines include a segment of the *P. falciparum* circumsporozoite protein (PfCSP), the most abundant protein expressed on the surface of sporozoites and in early liver forms. CSP plays an important role in sporozoite motility and entry into hepatocytes^6–8^. CSP contains three distinct regions: a conserved amino (N)-terminal region, a central region composed of NANP/NVDP repeats, and a carboxyl (C)-terminal region. RTS,S/AS01 and R21/MatrixM contain a truncated form of CSP with 19 NANP repeats from the central region and the C-terminal region that is fused to hepatitis B surface antigen (HBsAg) and expressed as virus-like particles in yeast^9^. The two vaccines differ in the ratio of HBsAg to the CSP protein with RTS,S having a fourfold excess of HBsAg relative to the CSP protein, and R21 has an expected 1:1 ratio^10^. Several novel approaches for second-generation CSP-based vaccines, such as protein nanoparticles^11^, mRNA^12,13^, self-replicating or replicon RNA (repRNA)^14^, DNA^15^, and tobacco mosaic virus (TMV) particles^16^ are under development. These vaccines target different regions of CSP that are recognized by the immune system and prevent liver invasion in animal models. The designed CSPj5c used here is comprised of the junction region (NPDPNANPNVDPNA), 5 NPNA repeats and the C-terminal segment that includes the TSR domains (310 to 383aa) of CSP. This design leverages the known epitope and protection data for CSP to include the B-cell epitopes for CIS43, mAb317, and L9 mAbs and the T-cell epitopes located in the C-terminal TSR domains^17^. CSP vaccines provide limited protection and partial efficacy with titers that wane over time necessitating booster doses to maintain protection. The limited protection allows some *Plasmodium* parasites to infect RBCs and morph into completely different life-cycle stages following breakthrough that can no longer be targeted by the vaccine and enabling disease transmission to the next human host. This problem can be ameliorated by combining a CSP vaccine with transmission-blocking vaccine that would prevent onward transmission in the event of breakthrough infection and reduce the parasite load in mosquitoes thereby reducing disease burden, transmission and severity in the human population^18^.

Pfs48/45 is one of the most promising targets for a malaria transmission blocking vaccine, but production challenges and limited potency have hampered vaccine progress over the last 40 years^19^. Pfs48/45 is expressed on the surface of gametocytes, plays an important role in the fertilization process^20^, and is known for its potential to induce potent antibodies that can block the transmission of malaria from humans to mosquitoes^21^. Pfs48/45 is a member of the six-cysteine protein family and contains N- and C-terminal 6-Cys domains (D1 and D3) joined by a central 4-Cys domain (D2). Both full-length Pfs48/45 and multiple domains are difficult to produce recombinantly, and numerous expression systems, such as *Escherichia coli*, baculovirus (*Spodoptera frugiperda* Sf9) cells, vaccinia virus, *Saccharomyces cerevisiae*, *Pichia pastoris*, *Chlamydomonas reinhardtii*, and *Nicotiana benthamiana*, have been evaluated with limited expression yields^22–27^. Full-length Pfs48/45 is a heavily glycosylated protein when expressed in a eukaryotic system, and it is challenging to produce correctly folded recombinant protein in a bacterial expression system^28^. Nevertheless, full-length Pfs48/45 has been successfully expressed in Drosophila S2 cells and is now in clinical trials (NCT05400746 & NCT06549257)^29^. Various fragments of Pfs48/45, such as 10 C (D2-D3) and 6 C (D3), fused to other antigens have been produced in *L. lactis* to improve the yield of correctly folded proteins and their immunogenicity^30–33^. R0.6 C and ProC6C have been evaluated in phase I clinical trials, eliciting detectable TRA in most participants, but only at 15 mg/mL IgG^34–36^. Additional development may be necessary to reach TRA levels required for clinical benefit.

Stabilizer for Protein Expression and Epitope Design (SPEEDesign) is a computational and in vitro screening pipeline^37^ that was used to develop an enhanced Pfs48/45 D3 immunogen (17-4) with improved stability, expression yields and immunogenicity, and that lacked glycosylation that hampered efficient production^38^. The presentation of 17-4 on ferritin nanoparticles further improved the immunogenicity and transmission-blocking activity^38^. Ferritin is a naturally occurring iron-bearing protein that self-oligomerizes or self-assembles 24-subunits in an octahedral symmetry and allows for higher-order presentation of one or more antigens on the surface of the nanoparticle^39,40^. Compared with single-subunit vaccines, the display of antigens on ferritin nanoparticles can enhance the immunogenicity of antigens, leading to long-lasting immunity. Previous studies have shown that compared with monomeric CSP, the CSP antigen on the surface of ferritin nanoparticles induces a significantly greater serum response^41,42^. *Helicobacter pylori* ferritin has been used to safely display antigens from a wide range of pathogens that are now in phase I clinical trials, such as influenza (NCT03186781, NCT03814720, and NCT04579250), Epstein-Barr virus (NCT04645147), and SARS-CoV-2 (NCT04784767)^43–46^.

Malaria vaccine development has primarily focused on the development of single antigens that are expressed at distinct stages of the malaria lifecycle. Fewer studies have focused on developing vaccine candidates with dual antigens from a single stage of malaria, such as CSP-TRAP^47^, Pfs48/45-230D1^33^, and Pb22-Pbg37^48^, and from different stages of malaria such as ProC6C^33^, CSP/Pfs230D1/CoPoP^49^, CSP-Pfs25^50^ and GMZ2.6c^51^. Genetic fusion of two proteins offers several advantages over combination vaccines that include enhanced immunogenicity, improved stability, safety and simplified production. Furthermore, safety, quality, efficacy, and immunogenicity may be affected by interactions between the antigens and other vaccine components when two or more independent vaccines are mixed^52,53^. In this study, we designed a potent malaria vaccine candidate that targets two different stages of the malaria parasite lifecycle by creating a chimeric antigen containing designed CSPj5c and Pfs48/45 17-4 immunogens. These antigens can be displayed on ferritin nanoparticles and immunization of mice and rabbits prevents both initial infection of the liver and transmission of the parasite to mosquito.

## Results

### CSPj5c and Pfs48/45 17-4 immunogen fusions can be expressed and purified from Expi293 mammalian cells

**Fig. 1:**
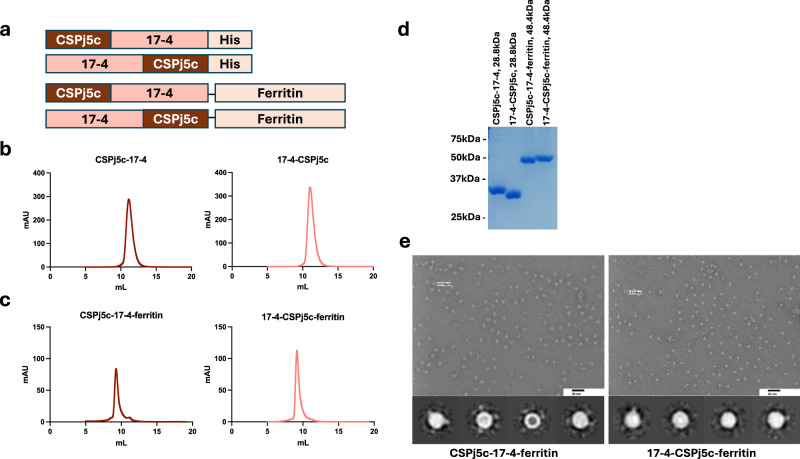
Designed antigens can be expressed and purified as monomers and displayed on ferritin nanoparticles. **a** Design of chimeric antigen as monomer and ferritin particle. CSPj5c and 17-4 are designed immunogens based on CSP and Pfs48/45 D3 proteins respectively. **b** Size-exclusion chromatography profile of CSPj5c-17-4 and 17-4-CSPj5c on Superose 6 Increase 10/300 GL column after nickel resin purification. **c** Size-exclusion chromatography profile of CSPj5c-17-4-ferritin and 17-4-CSPj5c-ferritin on Sepax SRT SEC-1000 column from Expi293F supernatant. **d** SDS-PAGE gel electrophoresis of purified monomeric protein and ferritin particle (reduced). **e** Negative-stain EM micrographs of purified nanoparticles displaying chimeric antigens with 2D classification averages (bottom) scale bar = 50 nm.

We designed a multistage malaria vaccine candidate by genetically fusing designed CSP and Pfs48/45 immunogens (Fig. 1a). Pfs48/45 17-4 (referred to as 17-4 hereafter) is a computationally designed immunogen that has been previously described to have higher yield, thermostability and functional activity than wild type D3^38^. CSPj5c is a designed CSP immunogen that contains the junction region (NPDPNANPNVDPNA), five NPNA repeats and the C-terminal TSR domain from residue 310 to residue 383. The CSPj5c immunogen was fused to either the N- or C-terminus of 17-4, and both orientations, as well as the individual CSPj5c and Pfs48/45-17-4 components, expressed in Expi293™ cells. The CSPj5c monomer elutes at ~12.7 mL on a Superdex 75 10/300 increase column (Cytiva) and can be observed as a single band of ~15 kDa on a reduced SDS-PAGE gel. CSPj5c migrated at a higher molecular mass as compared to the expected molecular weight (MW) of 13.1 kDa. This is consistent with other studies that have shown that CSP displays an aberrant electrophoretic mobility in SDS-PAGE gel^54,55^. The 17-4 monomer eluted on a Superdex 75 10/300 increase column at ~13.2 mL and appeared as a single band on an SDS-PAGE gel that is consistent with the theoretical molecular weight of 16.9 kDa (Supplementary Figs. 1 & 2). Both fusion proteins (CSPj5c-17-4 and 17-4-CSPj5c) eluted at ~11 mL on a Superdex 75 10/300 increase column (Fig. 1b). SDS-PAGE analysis revealed single band for the fusion monomers suggesting high purity although different migration patterns on reducing SDS PAGE gel were observed for the fusion monomers even though the expected molecular weight for both is 28.8 kDa (Fig. 1d).

### CSPj5c and Pfs48/45 17-4 fusion proteins fused to ferritin form homogenous nanoparticles

We additionally fused a ferritin subunit to our immunogens to create self-assembling nanoparticles. This ferritin fusion was previously shown to enhance the immunogenicity of 17-4 on its own^38^. CSPj5c-ferritin and 17-4-ferritin particles were purified on a Superose 6 column with elution volumes of ~12.2 mL and ~12.7 mL, respectively. Both samples were observed as a single band on an SDS-PAGE gel close to expected molecular weights of 32.9 kDa and 36.5 kDa, respectively (Supplementary Fig. 1 & 2). Both fusion ferritin particles (CSPj5c-17-4-ferritin and 17-4-CSPj5c-ferritin) were purified on Sepax SRT SEC-1000 column and eluted at ~9 mL consistent with the formation of a nanoparticle (Fig. 1c). Both fusion ferritin particles can be seen on an SDS-PAGE gel at ~ 50 kDa band consistent with the theoretical molecular weight of the monomeric unit of the particle (48.4 kDa) (Fig. 1d). Size-Exclusion Chromatography Small Angle X-ray Scattering (SEC-SAXS) and Multi-Angle Light Scattering (SEC-MALS) reported molecular weights consistent with the theoretical molecular weight of the monomer and of ferritin nanoparticle and fall within the limit of error for each technique (Table 1). These biophysical data indicate that fusion of CSPj5c and 17-4 did not hamper the production of designs.

**Table 1:** Molecular weight of monomeric fusion antigen and ferritin nanoparticle

Antigen	Theoretical molecular weight (kDa), Monomer	Theoretical molecular weight (kDa), Nanoparticle (24 mer)	Molecular weight (kDa) determined by SEC-MALS	Molecular weight (kDa) determined by SEC-SAXS	Radius of Gyration, R_g_ (Å) determined by SEC-SAXS
CSPj5c-17-4	28.8	-	27.9 ± 0.26	40.2	31.86 ± 0.49
17-4-CSPj5c	28.8	-	26.9 ± 0.17	36.9	30.23 ± 0.53
CSPj5c-17-4-ferritin	48.4	1162	911 ± 3.78	1200	85.03 ± 0.50
17-4-CSPj5c-ferritin	48.4	1162	954 ± 4.59	1200	88.71 ± 1.36

The theoretical molecular weights were calculated using Expasy ProtParam tool. The experimental molecular weight from SEC-MALS were determined using Wyatt ASTRA 8 software. Molecular weight from SEC-SAXS and the radius of gyration (R_g_) with standard deviation were determined using SIBYLS SEC-SAXS Process GUI and ATSAS software suite.

We evaluated the fusion ferritin particles by negative-stain electron microscopy (NS-EM) to establish if display of the antigens resulted in the correct nanoparticle architecture. Raw images and 2D class images revealed that pure and homogenous nanoparticles are formed with protruding visible densities around ferritin particle (Fig. 1e) consistent with the use of flexible linkers between the antigens and the ferritin nanoparticle.

### Fusion monomers and ferritin particles bind to CIS43 and TB31F mAbs

We probed the integrity and accessibility of neutralizing epitopes on both the CSPj5c and 17-4 components of our immunogens by using biolayer interferometry (BLI) with antibodies TB31F and CIS43 (Table 2 & 3 and Supplementary Figs. 3 & 4). TB31F is a highly potent humanized version of rat mAb 85RF45.1 that binds to Pfs48/45 protein^56^. TB31F inhibits gamete fertilization, prevents parasite development in the mosquito midgut and prevents parasite transmission^57^. CIS43 is a human monoclonal antibody that binds CSP repeats with a preference for the junctional NPDP region^58^. Both CIS43 and TB31F bound to the monomer designs in nanomolar affinity range and the ferritin nanoparticles with an apparent picomolar affinity range. Association rate (k_a_), dissociation rate (k_d_) and dissociation constant (K_D_) values are very similar for both N- and C- terminal CSPj5c fusions indicating that binding sites are largely conserved and accessible by both CIS43 and TB31F antibodies. Ferritin nanoparticles bound to the TB31F and CIS43 antibodies with lower association rates and negligible dissociation rates resulting in much lower K_D_ values. These data indicate that the epitopes are correctly presented on the nanoparticles, although accessibility may be reduced due to high-density display. This decrease in accessibility is offset by the decrease in dissociation, resulting in an increase in apparent affinity due to avidity induced by repetitive display of the antigens (Tables 2, 3).

**Table 2:** Binding kinetics and affinities to CIS43 antibody as determined by BLI

Antigen	K_D_(M) x 10^-9^	k_a_ (1/Ms) x 10^5^	k_d_ (1/s) x 10^-3^
CSPj5c	0.84 ± 0.36	22.4 ± 0.24	1.89 ± 0.27
CSPj5c-17-4	1.08 ± 0.08	14.6 ± 0.05	1.59 ± 0.16
17-4-CSPj5c	1.21 ± 0.09	13.1 ± 0.02	1.59 ± 0.13
**Antigen-ferritin**	**K**_**D**_**(M) x 10**^**-12**^	**k**_**a**_ **(1/Ms) x 10**^**5**^	**k**_**d**_ **(1/s) x 10**^**-7**^
CSPj5c-ferritin	1.80 ± 0.28	2.25 ± 0.03	4.08 ± 0.63
CSPj5c-17-4-ferritin	1.84 ± 0.27	2.32 ± 0.09	4.24 ± 0.46
17-4-CSPj5c-ferritin	1.88 ± 0.29	2.10 ± 0.03	3.94 ± 0.54

Dissociation constant (K_D_), association rate constant (k_a_), and dissociation rate constant (k_d_).

**Table 3:** Binding kinetics and affinities to TB31F antibody as determined by BLI

Antigen	K_D_(M) x 10^−^^10^	k_a_ (1/Ms) x 10^5^	k_d_ (1/s) x 10^−4^
17-4	7.70 ± 0.81	9.76 ± 0.60	7.66 ± 1.33
CSPj5c-17-4	6.26 ± 0.70	11.8 ± 0.01	7.44 ± 0.92
17-4-CSPj5c	6.21 ± 0.24	12.4 ± 0.01	7.74 ± 0.37
**Antigen-ferritin**	**K**_**D**_**(M) x 10**^**−12**^	**k**_**a**_ **(1/Ms) x 10**^**5**^	**k**_**d**_ **(1/s) x 10**^**−7**^
17-4-ferritin	2.95 ± 0.32	1.35 ± 0.05	3.96 ± 0.32
CSPj5c-17-4-ferritin	2.10 ± 0.26	1.98 ± 0.04	4.18 ± 0.49
17-4-CSPj5c-ferritin	1.55 ± 0.16	2.73 ± 0.06	4.23 ± 0.42

K_D_ Dissociation constant, k_a_ association rate constant k_d_ dissociation rate constant.

### Designs elicit strong immune responses to both antigens in C57BL/6 mice

**Fig. 2:**
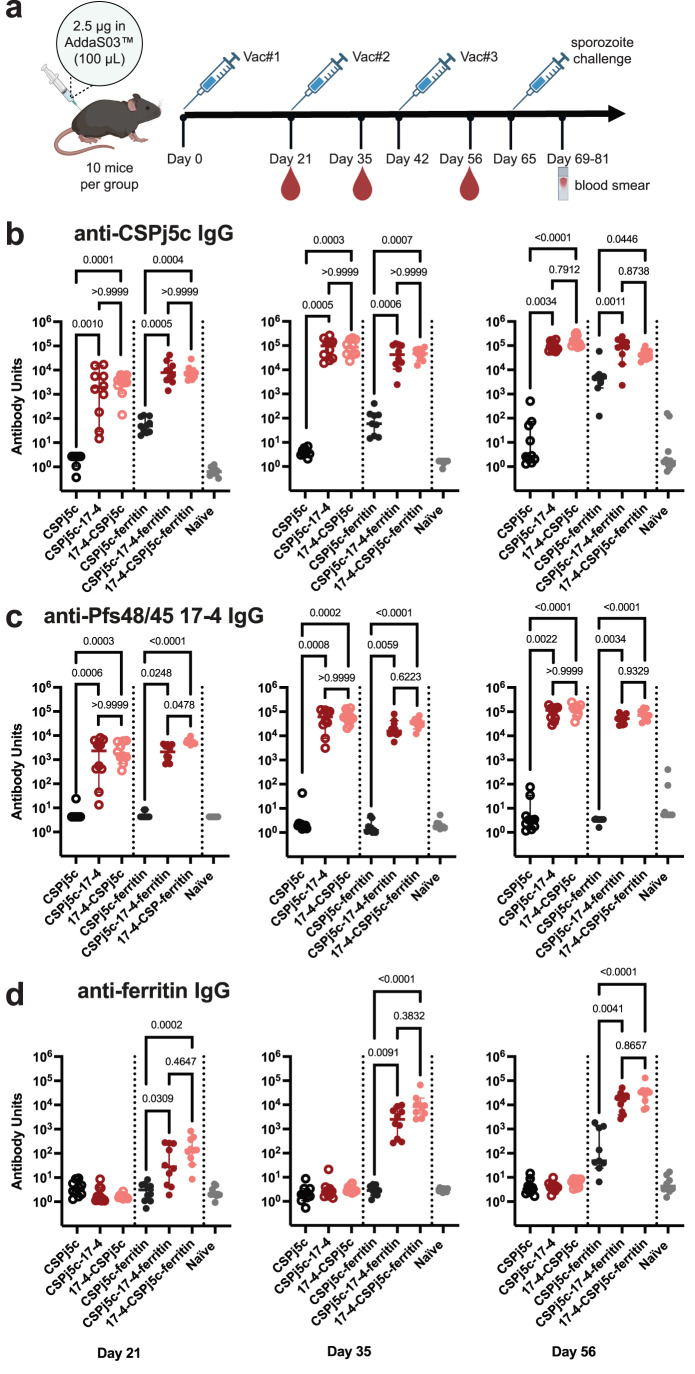
Designs elicit high antibody titers against target antigens in C57BL/6 mice. **a** Timeline for mouse immunization and sporozoite challenge (Created in BioRender. Dickey, T. (2025) https://BioRender.com/mcorhho). **b** IgG antibody titer against CSPj5c at days 21, 35 and 56. **c** IgG antibody titer against Pfs48/45 17-4 immunogen at days 21, 35 and 56. **d** IgG antibody titers against ferritin at days 21, 35, and 56. For all panels, median values and 95% confidence levels are displayed with individual values, and the *p*-values were calculated using the Kruskal-Wallis non-parametric test followed by Dunn’s test corrected for the three comparisons in each section of the graph.

The immunogenicity of fusion protein monomers and the ferritin nanoparticles were evaluated in C57BL/6 mice. A three-vaccination study was performed in C57BL/6 mice with a 2.5 ug dose of antigen formulated in AddaS03 injected at days 0, 21 and 42. Sera collected at days 21, 35 and 56 were assayed by ELISA to measure antibody response to CSPj5c, Pfs48/45 17-4 immunogen, ferritin (Fig. 2), and Pf48/45 WT (Supplementary Fig. 5). High IgG antibody titers to CSPj5c, Pfs48/45 17-4 and Pf48/48 WT were induced by fusion proteins and the fusion ferritin particles (Fig. 2b−d, Supplementary Fig. 5, Supplementary Tables 2 & 3). Titers in these groups were significantly greater than the naïve group after one or two immunizations and generally increased after each vaccination (Supplementary Table 2).

We performed statistical comparisons between the monomeric proteins to determine if fusion of additional antigens affected immunogenicity and if the orientation mattered (Fig. 2b, c). Strikingly, addition of 17-4 to either the N- or C- terminus of CSPj5c significantly increased the CSPj5c-specific titers at all time points (Fig. 2b). There was no significant difference in CSPj5c titers between the 17-4-CSPj5c and CSPj5c-17-4 groups, indicating that the 17-4-induced increase in immunogenicity was independent of orientation in the monomers (Fig. 2b). In addition, there was no significant difference in Pfs48/45 titers between the 17-4-CSPj5c and CSPj5c-17-4 groups, consistent with orientation-independent CSPj5c titers for the monomers in mice (Fig. 2c).

We also performed statistical comparisons between the ferritin groups to determine if fusion and orientation affected immunogenicity (Fig. 2b, c). Like the monomers, fusion of 17-4 to either the N- or C-terminus of CSPj5c significantly increased the immunogenicity of CSPj5c at all time points (Fig. 2b). Titers against CSPj5c were not significantly different between the two orientations (Fig. 2b) consistent with the observation that the 17-4-induced immunogenicity increase in CSPj5c titers is independent of orientation. When 17-4 was on the exterior of the nanoparticle (17-4-CSPj5c-ferritin) titers to 17-4 were significantly higher than the other orientation after one immunization, but this difference was not significant after additional immunizations (Fig. 2c). Titers against ferritin were significantly higher in the fusion groups than CSPj5c-ferritin suggesting that fusion increases the overall immunogenicity of the nanoparticle (Fig. 2d). Together, these results suggest that fusion of 17-4 increases CSPj5c immunogenicity and that the orientation of the fusion does not substantially impact the immunogenicity of either antigen.

### Designs confer sterile protection upon PbPfCSP sporozoite challenge in C57BL/6 mice

We assessed protective efficacy of the fusion protein monomers and the ferritin particles upon challenge with PbPfCSP sporozoites. Immunized mice were challenged with 500 PbPfCSP sporozoites by intradermal administration at day 65 and the presence of blood infection was evaluated up to 12 days post-challenge. Three groups (CSPj5c-17-4, CSPj5c-17-4-ferritin, and 17-4-CSPj5c-ferritin) provided significant protection relative to naïve mice (Supplementary Table 4).

**Fig. 3:**
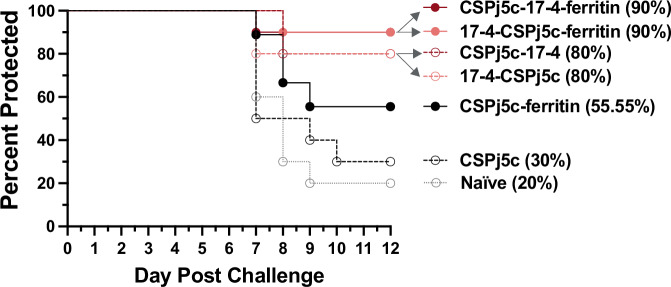
Designs confer sterile protection against sporozoite challenge. Protection of C57BL/6 mice from infection after challenge with 500 PfCSP transgenic *P. berghei* parasites. The statistical significance of differences in survival analysis was determined via Kaplan‒Meier survival analysis with Bonferroni correction for multiple comparisons.

We next investigated if addition of 17-4 to CSPj5c improved protection conferred by the monomers. Consistent with ELISA results, addition of 17-4 to either terminus of CSPj5c increased protection (Fig. 3). Monomeric CSPj5C conferred 30% sterile protection while both N- and C-terminal 17-4 fusions reached 80% protection. This enhancement is statistically significant after Bonferroni correction for two comparisons for CSP-17-4 (*p* = 0.0223) and just above the *p* < 0.025 threshold for 17-4-CSP (*p* = 0.0306).

We further examined if addition of 17-4 to CSPj5c, when presented on ferritin nanoparticles, improved protection. Fusion of 17-4 to either the N- or C-terminus of CSPj5c increased protection from 56% (CSPj5c-ferritin) to 90% (CSPj5c-17-4-ferritin and 17-4-CSPj5c-ferritin). These enhancements are not statistically significant (*p* = 0.0935 and 0.1148) with ten mice per group, but become significant if the fusion groups are combined post hoc to specifically address the question if fusion improves activity (*p* = 0.0005). The consistent trend of fusion proteins being more immunogenic and protective provides strong evidence that 17-4 fusion enhances CSPj5c immunogenicity and protectivity.

Nanoparticle display of the antigens also consistently increases protection. CSP-ferritin achieves greater protection (56%) than CSPj5c (30%) and the fusion protein nanoparticles achieve greater protection (90%) than the monomers (80%), although these differences are not statistically significant. Together, these results suggest that fusion of 17-4 increases the protection conferred by CSPj5c and nanoparticle display can further increase this protection.

### Designs elicit strong immune responses to both antigens in rabbits

**Fig. 4:**
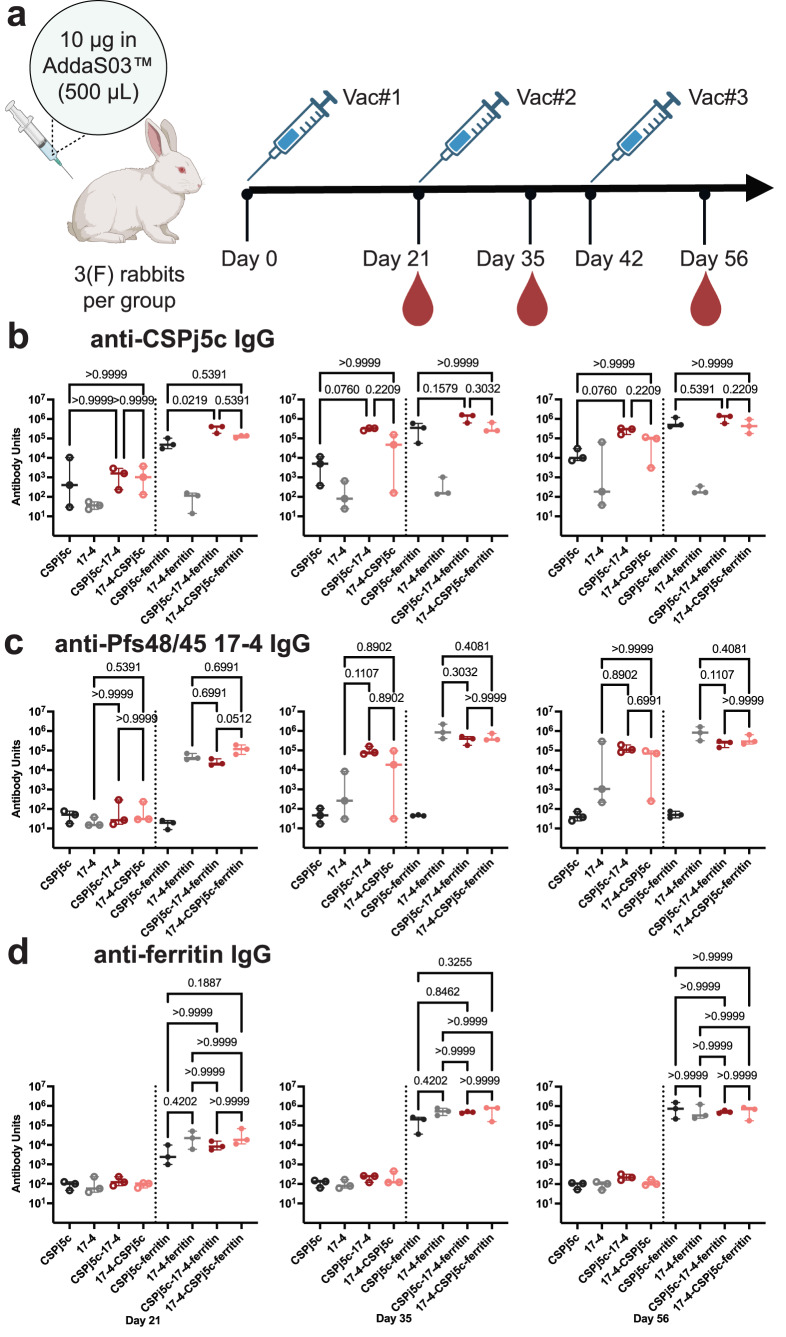
Designs elicit high antibody titers against target antigens in rabbits. **a** Timeline for rabbit immunization (Created in BioRender. Dickey, T. (2025) https://BioRender.com/rf31hos). **b** IgG Antibody titers to CSPj5c at days 21, 35 and 56. **c** IgG Antibody titers to Pfs48/45 17-4 immunogen at days 21, 35 and 56. **d** IgG antibody titers to ferritin at days 21, 35, and 56. For all panels, median values and 95% confidence levels are displayed with individual values, and the *p*-values were calculated using Kruskal-Wallis non-parametric test with Dunn’s multiple comparisons.

The immunogenicity of the monomers and the ferritin particles were also evaluated in a second species by immunization of New Zealand white rabbits. Rabbits were immunized three times at three weeks interval with 10 µg of antigens formulated in AddaS03. Sera collected at days 21, 35 and 56 were assayed by ELISA to measure the antibody response against CSPj5c, Pfs48/45 17-4 immunogen, ferritin (Fig. 4), and Pfs48/48 WT (Supplementary Fig. 6). Antibody titers increased after each vaccination in both monomer and ferritin nanoparticle groups (Fig. 4 and Supplementary Tables 5, 6). The ferritin titers are roughly proportional to the antigen titers suggesting that antigens do not differentially mask the nanoparticle. As in mice, the fusion monomers elicited higher CSPj5c specific titers than the CSPj5c monomer, suggesting that 17-4 increases CSPj5c immunogenicity, however, these differences are not significantly different with only three animals per group. At day 56, CSPj5c-17-4 orientation elicited slightly higher titers than 17-4-CSPj5c in the monomer, although this difference is not statistically significant with three animals. This trend is true for both CSP specific and Pfs48/45 specific antibodies suggesting that the CSPj5c-17-4 orientation could be more stable or immunogenic.

### Potent transmission reducing activity elicited by fusion antigens on ferritin nanoparticles

**Fig. 5:**
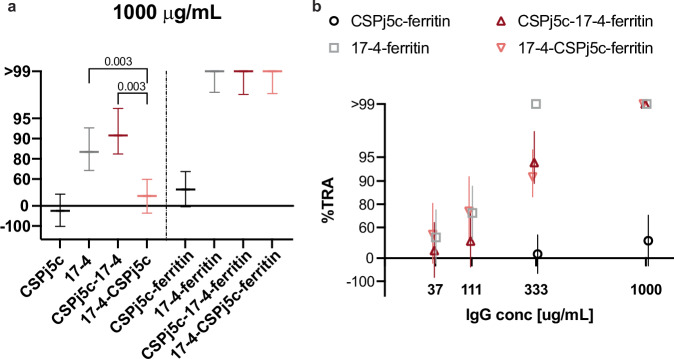
Potent transmission reducing activity elicited by fusion antigens on ferritin nanoparticles. Transmission reducing activity by SMFA with purified IgG from pooled sera of immunized rabbits. **a** The IgG for each group at 1000 μg/mL was tested in two separate SMFAs, and the transmission reducing activity (TRA) is shown as solid lines with 95% CI (error bars). The 95%CI and statistical significance were calculated by the zero-inflated negative binomial model (ZINB model) with Bonferroni corrections. **b** Ferritin nanoparticle groups were tested at lower IgG concentrations to quantitatively compare their potency. Symbols indicate TRA values with 95% CI error bars. Data at 1000 and 333 μg/mL come from two separate SMFAs, and all other data comes from a single feed.

The transmission blocking potential of the designs were evaluated in the standard membrane feeding assay (SMFA) for transmission reducing activity (Fig. 5). SMFA was performed using purified IgG from pooled sera at two concentrations (333 μg/mL and 1000 μg/mL). The 17-4-containing immunogens CSPj5c-17-4 monomer, CSPj5c-17-4-ferritin, 17-4-CSPj5c-ferritn, and 17-4 ferritin elicit significant TRA at 1000 μg/mL (Fig. 5a and Supplementary Table 7). Only the 17-4-CSPj5c monomer did not demonstrate significant TRA at 1000 μg/mL. At 333 μg/mL IgG, only the 17-4-containing ferritin groups demonstrated significant TRA, while the monomers did not (Supplementary Table 7).

Next, we compared the TRA elicited by the monomer and the respective ferritin nanoparticle within the same immunogen group at each concentration. For all immunogens except CSPj5c, fusion of the monomer to ferritin particles induced significantly higher %TRA (*p* = 0.001) (Supplementary Tables 7 & 8). Therefore, ferritin-nanoparticle display significantly improved transmission reducing activity.

We further examined if addition of CSPj5c to 17-4 had any effect on %TRA within monomers and the ferritin particles, and if %TRA reflects any orientation bias with N-terminal or C-terminal fusion of 17-4 to CSPj5c (Fig. 5 & Supplementary Tables 7 & 9). Within monomers, %TRA at 1000 μg/mL suggests that fusion of CSPj5c to the C-terminus of 17-4 (17-4-CSPj5c) reduced the TRA significantly (38.9%TRA) when compared to 17-4 alone (84.2%TRA) or CSPj5c at the N-terminus (CSPj5c-17-4 91.0%TRA) (*p* = 0.003). CSPj5c-17-4 (91% TRA) did not demonstrate a significant difference to 17-4 monomer (84.2% TRA) (*p* = 0.546). These data suggest orientation bias in inducing TRA and that the CSPj5c-17-4 orientation retains TRA similar to 17-4 alone.

To better quantify the difference between the ferritin nanoparticles we performed additional SMFAs with IgG at 333, 111, and 37 μg/mL (Fig. 5b). The addition of CSPj5c to either terminus significantly reduced the %TRA at 333 μg/mL when compared to 17-4-ferritin (*p* = 0.003) although the magnitude of the difference was small (CSPj5c-17-4-ferritin: 94.2%, 17-4-CSPj5c-ferritin: 91.2%, 17-4-ferritin: 100.0%). At 111 and 37 μg/mL, all groups had < 80% TRA and were not significantly different. It should be noted that the minor significant reduction in TRA observed at 333 μg/mL may be an artifact of the lower molar dose of 17-4 received by these animals. Animals received equal mass of antigen (10 μg) meaning addition of CSP to 17-4-ferritin results in a molar dose of 17-4 that is 76% that of 17-4-ferritn. This lower molar dose in the CSP-17-4-ferritin and 17-4-CSP-ferritin groups as compared to 17-4-ferritin may account for the less than 10% point difference observed at the 333 μg/mL concentration. There was not a consistent difference between CSPj5c-17-4-ferritin and 17-4-CSPj5c-ferritin suggesting that orientation does not significantly affect the activity of the nanoparticles. In summary, 17-4-containing nanoparticles elicit potent TRA, and the addition of CSPj5c does not reduce TRA activity by a large magnitude.

## Discussion

In this study, we have generated a malaria vaccine candidate with pre-erythrocytic (CSPj5c) and transmission blocking (Pfs48/45 17-4) vaccine antigens that are genetically fused and displayed as single-component self-assembled ferritin nanoparticles. We successfully purified both the two antigen fusion proteins and the equivalent fusion ferritin nanoparticles using a mammalian expression system that allows for proper disulfide bond formation and post-translational modifications. The designed fusion proteins are properly folded as the neutralizing epitopes present in CSPj5c and 17-4 are recognized by both the CSP-specific CIS43 antibody and the Pfs48/45-specific TB31F antibody.

Fusion of two antigens may result in a misfolded protein and can affect the immunogenicity of either antigen. We evaluated if immunogenicity is affected upon fusion of two antigens in mice and rabbits and demonstrated that fusion of the two antigens did not significantly hamper the immunogenicity of either antigen. Strikingly, the addition of 17-4 to CSPj5c enhanced the immunogenicity of CSPj5c eliciting significantly higher antibody response as compared to monomeric CSPj5c and resulting in better CSP-mediated protection against sporozoite challenge. Monomeric CSPj5c was poorly immunogenic likely due to its small size as immunogen size has a direct impact on immunogenicity^40^, and the addition of the computationally stabilized 17-4 likely improved immunogenicity by increasing stability as well as immunogen size. Similarly, addition of Pfs230D1 to a CSP-CoPoP nanoparticle increased immunogenicity and protection, suggesting the addition of stable domains may improve CSP vaccines^49^. However, the Pfs230D1 enhancement was seen only when comparing groups that received equimolar amounts of CSP. Here, we see an enhancement even when equal masses of vaccine are delivered and the fusion protein contains reduced molar quantities of CSPj5c. Additionally, a recent study showed that the addition of the native CSP sequence N-terminal to CSPj5c, which is likely unstructured, reduced titers and protection, meaning that the fusion partner must be chosen carefully^59^. This suggests that 17-4 has specific beneficial effects on CSPj5c, likely due to increasing stability of the fusion, and can improve CSP-mediated protection from infection by sporozoites.

Antigen orientation on nanoparticles has been shown to influence the immunogenicity and protective efficacy of displayed antigens. SAPN displaying PfCelTOS on the C-terminus of MSP1-19 had higher PfCelTOS-specific antibody as compared to N-terminal localization^60^. Similarly, when TRAP was fused to the N-terminus of a CSP, but not the C-terminus, the protein had high production yields and conferred durable, as well as, sterile protection against *P. berghei* infection in BALB/c mice^47^. We also investigated the effect of antigen orientation in the monomeric fusion proteins and ferritin particles on immunogenicity and protective efficacy. CSPj5c at the N- terminus of 17-4 monomer had better transmission reducing activity as compared to CSPj5c at the C- terminus of 17-4 in the rabbit study, but this difference was not significant for the nanoparticles. Additionally, no major differences in immunogenicity, as measured by antibody titer, in either mice or rabbits was observed for the different orientations in the monomer or ferritin particles. Further, similar protective responses against sporozoite challenge by N- or C- terminal fusion of CSPj5c to 17-4 in mice were observed. Together the data indicate that orientation can have an impact on certain outcomes.

While this study establishes an effective combined infection-blocking and transmission-blocking vaccine the limitations of this study are that the long-term protective efficacy, dose finding studies, adjuvant evaluation of the CSPj5c-17-4-ferritin, and linker length between the CSPj5c and 17-4 have not been investigated. The promising protective data of CSPj5c-17-4-ferritin established in a mouse model of malaria and in rabbits forms a strong foundation for the further evaluation in non-human primates that is imperative to inform the development of vaccine for human use. Furthermore, the mechanism for enhanced infection blocking activity imparted by the addition of 17-4 has not been established. Future studies will address these key points towards a deeper understanding of protection and establishment of a combined infection and transmission blocking vaccine. An additional limitation is that there is no direct comparison to existing CSP-based or Pfs48/45-based vaccines to evaluate the predictive power of the designs developed here. A recent study tested several different CSP designs and found that a design similar to CSPj5c (CSP K) provided significant protection when displayed on a nanoparticle^59^. The level of protection elicited by the CSP K nanoparticle, however, was slightly lower than the approved R21 vaccine. We expect that our CSPj5c-ferritin would confer protection similar to the CSP K nanoparticle, and that the enhanced protection conferred by CSPj5c-17-4-ferritin may reach levels comparable to R21. In addition, CSPj5c-17-4-ferritin has the unique ability to elicit potent transmission-blocking activity. Direct comparisons are likely to be highly informative and will be investigated in future studies that are now feasible given the establishment of CSPj5c-17-4-ferritin presented here.

This study demonstrates that the CSPj5c-17-4-ferritin particle elicits potent infection- and transmission-blocking activity. This activity is achieved with a single genetically fused protein which greatly simplifies manufacturing, dosing, and administration compared to a mixture of two antigens or two separate vaccines. The 90% protection we observe is promising, but it is imperfect, as seen with RTS,S, R21, and many other vaccines targeting diverse pathogens. However, this dual-stage vaccine also elicits transmission-blocking activity that could limit onward transmission. TBVs on their own do not protect the individual receiving the vaccine and it is possible that a stand-alone TBV could face regulatory and uptake challenges as a result. CSPj5c-17-4-ferritin addresses these concerns by protecting the vaccinee from infection and additionally protecting the community by reducing onward transmission. In combination with other antigens, vaccines, and interventions, this vaccine could contribute to the long-sought goal of eradication.

## Methods

### Antigen design, expression, and purification

Antigen designs are summarized in Fig. 1a. and sequence data provided in Supplementary Table 1. The Pfs48/45 17-4 immunogen (referred to as 17-4) has been previously described in ref.^38^. The CSPj5c immunogen contains junction region (NPDPNANPNVDPNA), five NPNA repeats and the C-terminal TSR domain from residue 310 to residue 383. Pfs48/45 17-4 is fused either at the N-terminal or C-terminal of CSPj5c to generate fusion proteins (CSPj5c-17-4 and 17-4-CSPj5c). Sequences were synthesized and subcloned into pHLSec expression vector (GenScript, a gift from Edith Yvonne Jones (Addgene plasmid # 99845; http://n2t.net/addgene:99845; RRID: Addgene_99845)). Plasmids were transiently transfected into Expi293F cells following manufacturer’s instructions (Thermo Fisher Scientific). Supernatant was harvested after 4 days of expression at 37 °C. Soluble protein was purified using Ni Sepharose excel resin (Cytiva,17371203) and then by size-exclusion chromatography using a Superdex 75 Increase 10/300 GL column (Cytiva, 29148721) equilibrated in phosphate buffered saline (1x PBS). Peak fractions were pooled, flash frozen in liquid nitrogen, and stored at −80 °C.

Antigen-ferritin nanoparticles were created by genetically fusing the fusion proteins (CSPj5c-17-4-ferritin and 17-4-CSPj5c-ferritin) and the immunogens (CSPj5c-ferritin and 17-4-ferritin) to N-terminus of the ferritin with a GSGGGG linker. The ferritin construct is a genetically engineered *Helicobacter pylori*-bullfrog hybrid ferritin that has been described previously^61^. Ferritin nanoparticle containing the fusion proteins, or the immunogens were expressed in Expi293F cells, supernatant was harvested after 4 days of expression at 37 °C and concentrated using 100 kDa molecular weight cutoff Amicon centrifugal filter units. Concentrated supernatant was purified by size-exclusion chromatography using a SRT SEC-1000 column (Sepax Technologies, 215950-7830) or Superose 6 Increase 10/300 GL (Cytiva, 29091596) equilibrated in 1x PBS. Peak fractions were pooled, flash frozen in liquid nitrogen, and stored at −80 °C. Cloning, expression, and purification of TB31F and CIS43 humanized mAb was performed as described previously^38^.

### Molecular weight determination by SEC-MALS and SEC-SAXS

SEC-MALS and SEC-SAXS were conducted at SIBYLS at beamline 12.3.1 at the Advanced Light Source^62–64^. This beamline has additional inline instruments and detectors coupled to a size exclusion chromatography column. Purified fusion proteins were loaded onto pre-equilibrated (1× PBS, pH 7.4) Shodex PROTEIN KW-802.5 SEC column (Resonac Corporation, Tokyo, Japan) and the fusion ferritin particles were loaded onto a PROTEIN KW-804 SEC column (Resonac Corporation). SEC-MALS was performed on an Agilent 1260 series HPLC coupled with Wyatt Dawn Helos multi-angle light scattering (MALS) and Optilab refractive index detectors. Data was analyzed and molecular weights were determined using Astra 8 software package (Wyatt Technology). SEC-SAXS was performed on an Agilent 1260 series HPLC coupled with SAXS flow cell. The data was collected at a wavelength of 1.03 Å and at sample to detector distance of 1.5 m. A series of 3 s exposures were collected for each frame over the course of 40 min. Data for SEC-SAXS was analyzed by SIBYLS SEC-SAXS Process GUI and ATSAS software suite^65^.

### Nanoparticle evaluation by negative-staining EM

Purified antigen-ferritin nanoparticles (0.02 mg/ml) were adsorbed on a 300-mesh carbon-coated copper grid (EMD Science) for 30 s followed by NanoW (Nanoprobes) staining. Raw micrographs were recorded on Thermo Scientific Tecnai T20 microscope equipped with a charge-coupled device (CCD) camera. Particles in the images were auto picked using Gautomatch (http://www.mrc-lmb.cam.ac.uk/kzhang/) and 2D classes were generated using RELION 3.0^66^.

### Neutralizing antibody binding by Biolayer interferometry (BLI)

Binding affinity of designed antigens and antigen-ferritin nanoparticles to the TB31F and CIS43 antibody was measured by kinetic experiments performed on an Octet RED96e (Sartorius). Purified antigens, TB31F, and CIS43 were buffer exchanged into 1X HBS-EP buffer (10 mM HEPES (pH 7.4), 150 mM NaCl, 3 mM EDTA, 0.005% v/v surfactant P20, Cytiva, BR100826) using 7 K MWCO Zeba spin desalting columns (Thermo Fisher Scientific, 89877). BLI experiments were performed at a concentration of 125 nM for each design and 3 nM for TB31F and CIS43 antibody. All assay steps were performed in a 96-well black plates (Greiner Bio-One, 655209) at final volume of 200 µl at 25 °C. Anti-Human IgG Fc capture biosensors (Sartorius, 18-5060) tips were hydrated and equilibrated with 1X HBS-EP buffer for 600 s. Baseline was setup for 180 s and then purified antibody (3 nM) was immobilized on the biosensor tips for 300 s. Tips were washed, a second baseline was established for 60 s before measuring association kinetics for 300 s. For association kinetics measurement, designed antigens were 2-fold serially diluted in HBS-EP buffer. Finally, dissociation kinetics were measured for 300 s or 1200 s. Background subtraction was performed using an antibody-loaded sensor tip submerged into a well containing buffer only. Raw data was processed, association rate (k_a_), dissociation rate (k_d_), and dissociation constant (K_D_) were obtained by global fitting to a 1:1 binding model in Data Analysis HT12.0 software (Sartorius). Measurements were performed in three replicates with values reported as an average and SEM between replicates.

### Animal ethics statement

All animal studies were reviewed and approved under protocol LMIV 1E by the Institutional Animal Care and Use Committee (IACUC) at the National Institutes of Health (approval code: LMIV 1E and approval date: 10/1/2024) and performed in an American Association for Accreditation of Laboratory Animal Care (AAALAC)-accredited facility (AAALAC file # 000777, last accredited in 2021). The PHS Animal Welfare Assurance (File Number # D16-00602) was last approved 30 May 2023. Studies were designed to reduce animal numbers where possible, and no more than momentary pain or distress was anticipated. All housing, husbandry practices, and pain management were in accordance with AAALAC guidelines, standards, and regulations.

### Immunization and sporozoite challenge study in C57BL/6 mice

Five- to six-week-old C57BL/6 mice (Charles River Laboratories) were subcutaneously immunized with 2.5 μg of antigen on day 0, day 21, and day 42. Antigens were formulated in a 1:1 volume ratio in AddaS03™ Adjuvant (InvivoGen, vac-as03-10) with 100 μl of formulated antigen delivered by subcutaneous injection in one site in inguinal area. Naïve mice were used as a control group. Each group contained ten mice. Sera was collected and stored at −80 °C on day 21, day 35, and day 56 for analysis. One mouse in CSPj5c-ferritin group died and the death was determined not related to immunization. In addition, day 56 sera for a single mouse in 17-4-CSPj5c-ferritin group spilled during transfer.

Pb PfCSP 2257 SPZ (transgenic Pb ANKA line 2257, PfCS(r)PbCS GFP::LucPbeef1a - a gift from Dr. Chris Janse, Leiden University Medical Center)^67,68^ sporozoites expressing Plasmodium falciparum (Pf) CSP were prepared through cyclic transmission in BALB/c mice and Anopheles stephensi mosquitoes at the Laboratory of Malaria Immunology and Vaccinology (LMIV) Insectary, National Institutes of Health (NIH) in Bethesda, MD, USA. The growth cycle began by inoculating mice with blood-stage Pb PfCSP 2257 parasites, which were then used to feed female Anopheles stephensi mosquitoes. Mosquito salivary gland sporozoites (SPZ) were isolated and harvested 18-27 days post blood meal, as previously described^69^. Briefly, infected mosquitoes were dissected to remove their salivary glands, which were then placed in 0.3 ml of 0.22 μm filter-sterilized medium E199 (Quality Biologicals, 112-022-101) containing 0.2% bovine serum albumin (MP Biomedicals, 160069) in 1.5 ml Protein LoBind® Tubes (Eppendorf, 022431081). The salivary glands were triturated 20 times using a 1.0 ml syringe (BD, 309701) and a 26 G needle (BD, 305110) to release the SPZ. The SPZ were counted using a disposable hemocytometer (INCYTO, DHCN015), and their count per ml was determined. All study and control mice were intradermally (ID) challenged at the base of the tail at two sites with live SPZ using a 29 G 1.0 ml insulin syringe (Exelint, 26028), administering 500 SPZs in 100 μL of E199 containing 0.2% BSA (50 μL per site) to ensure reliable infection of all mice. Breakthrough to blood-stage infection was assessed by Giemsa-stained thin blood smears from day 4 to day 12 post-challenge. A negative smear by the end of this period was considered indicative of sterile protection. Mice were considered positive if infected red blood cells (iRBCs) were detected in more than one out of twenty random fields of view per blood smear. Mice with three consecutive days of positive parasitemia were euthanized, and a final blood smear was collected. Mice that remained negative for blood-stage infection were euthanized at the end of the study, either on day 12 or 14 post-challenge. Prior to euthanasia, mice were anesthetized via intraperitoneal injection of 125 mg/kg ketamine + 10 mg/kg; upon full sedation, blood samples were collected and euthanasia was performed by cervical dislocation.

### Rabbit immunization and Standard Membrane Feeding Assay (SMFA) for Transmission Reducing Activity (TRA)

Approximately eleven-week-old female New Zealand White rabbits (Charles River Laboratories) were subcutaneously immunized with 10 μg of antigen on day 0, day 21, and day 42. Each group contained three rabbits. Antigens were formulated in a 1:1 volume ratio in AddaS03™ Adjuvant (InvivoGen) with 500 μl of formulated antigen delivered by subcutaneous injection in one site in the dorsal area. Sera was collected and stored at −80 °C on day 0, day 21, day 35, and day 56 for further analysis. At end of study, rabbits were anesthetized via intramuscular injection with 0.1 mg/kg Dexdomitor® + 25 mg/kg ketamine, followed by 1.5 mL of phenobarbital euthanasia solution.

Functional activity of immunized rabbit sera was measured by SMFA as described previously^70^. Sixty μL of purified IgG from pooled sera were mixed with 200 μL of gametocyte mixture containing ~0.2% of stage V gametocytes at 50% hematocrit (*P. falciparum* NF54 strain), normal human serum and RBCs. SMFA was performed at the indicated concentration of purified IgG (1000, 333, 111, or 37 μg/mL). The mixture is then fed to 3-6-day old female *Anopheles stephensi* mosquitoes through a membrane feeding apparatus. The mosquitoes were maintained for 8 days and then dissected to count the number of oocysts per midgut in 20 blood-fed mosquitoes. %TRA was calculated as: 100 × [1 − (mean number of oocysts in the test group) / (mean number of oocysts in the control groups)]. TRA values at 1000 and 333 μg/mL are reported from two independent feeds at each concentration, while 111 and 37 μg/mL data came from a single feed.

### Determination of serum antibody titer by ELISA

Nunc MaxiSorp flat-bottom 96-well ELISA plates (Thermo Fisher Scientific, 44-2404-21) were coated with 100 μl of 0.05 mg/ml purified Pfs48/45 D3 WT, Pfs48/45 17-4 immunogen or CSPj5c diluted in 50 mM Na-carbonate pH 9.5, overnight at 4 °C. Plates were blocked for 1 h at room temperature with 2% bovine serum albumin (BSA) in phosphate buffered saline (PBS) containing 0.05% Tween 20 (PBST). Sera from each animal was diluted in PBST + 2% BSA and incubated for 1 h at room temperature. Anti-mouse IgG HRP-conjugated (Jackson ImmunoResearch, 115-035-164) or anti-rabbit IgG HRP conjugated (Bethyl, A120-111P) was added to each well at 1:10,000 or 1:5,000 dilution respectively and incubated for 30 min at room temperature. Finally, after incubation with secondary antibody, plates were washed three times with PBST and developed with 70 μl of TMB substrate. Reaction was stopped by adding 0.16 M sulfuric acid (H_2_SO_4_) and absorbance at 450 nm was measured using a BioTek™ Synergy H1 microplate reader.

Antibody titers of individual animals in all groups were measured against a standard curve on each plate. Serum from immunized mice or rabbits were pooled to generate the standard curve. One antibody unit (AU) was defined as the dilution of the standard serum required to achieve an Abs450 value of 1. Each plate included a triplicate 2-fold serial dilutions of the standard serum from 20 to 0.01 AU. Abs450 values of standard curve were fit to a 4-parameter logistic curve, which was used to convert Abs450 values of each individual animal to antibody units. AU values for each animal were measured in triplicate and the average values are reported.

## Supplementary information

Supplementary Information

## Data Availability

All data needed to evaluate the conclusions in the paper are present in the paper and/or Supplementary Materials. Plasmids can be provided by N.H.T. pending scientific review and a completed material transfer agreement. Requests should be submitted to N.H.T.
