# Supplementary material for: A combined designed CSP and Pfs48/45 infection and transmission blocking vaccine for malaria
Source: NPJ Vaccines. 2025 Sep 2;10:208. doi: 10.1038/s41541-025-01262-2 (PMC12405497; doi:10.1038/s41541-025-01262-2)
Supplement: Supplementary file 1 — Supplementary Information [file 41541_2025_1262_MOESM1_ESM.pdf]

Supplementary Information for

A combined designed CSP and Pfs48/45 infection- and transmission blocking- vaccine for malaria.

Richi Gupta, Thayne H. Dickey, Nichole D. Salinas, Palak N. Patel, Rui Ma, Dashuang Shi, Myesha Singleton, Tarik Ouahes, Thao P. Pham, Kazutoyo Miura, Carole A. Long, Lynn E. Lambert, and Niraj H. Tolia

Corresponding author email: niraj.tolia@nih.gov

This PDF file includes:

Supplementary Figures 1 to 6
Supplementary Tables 1 to 9

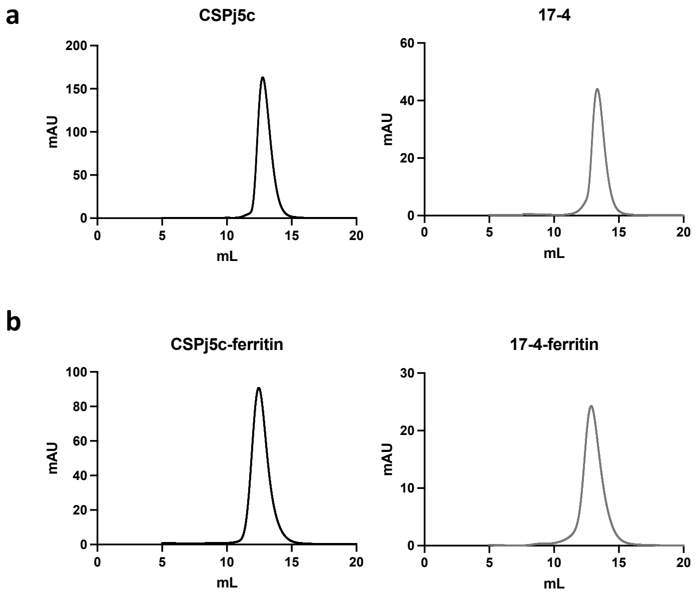

Supplementary Figure 1. a) Size-exclusion chromatography profile of CSPj5c and 17-4 on Superdex 75 Increase 10/300 GL column after nickel resin purification. b) Size-exclusion chromatography profile of CSPj5c-ferritin and 17-4-ferritin on Superose 6 Increase 10/300 GL column from Expi293F supernatant.

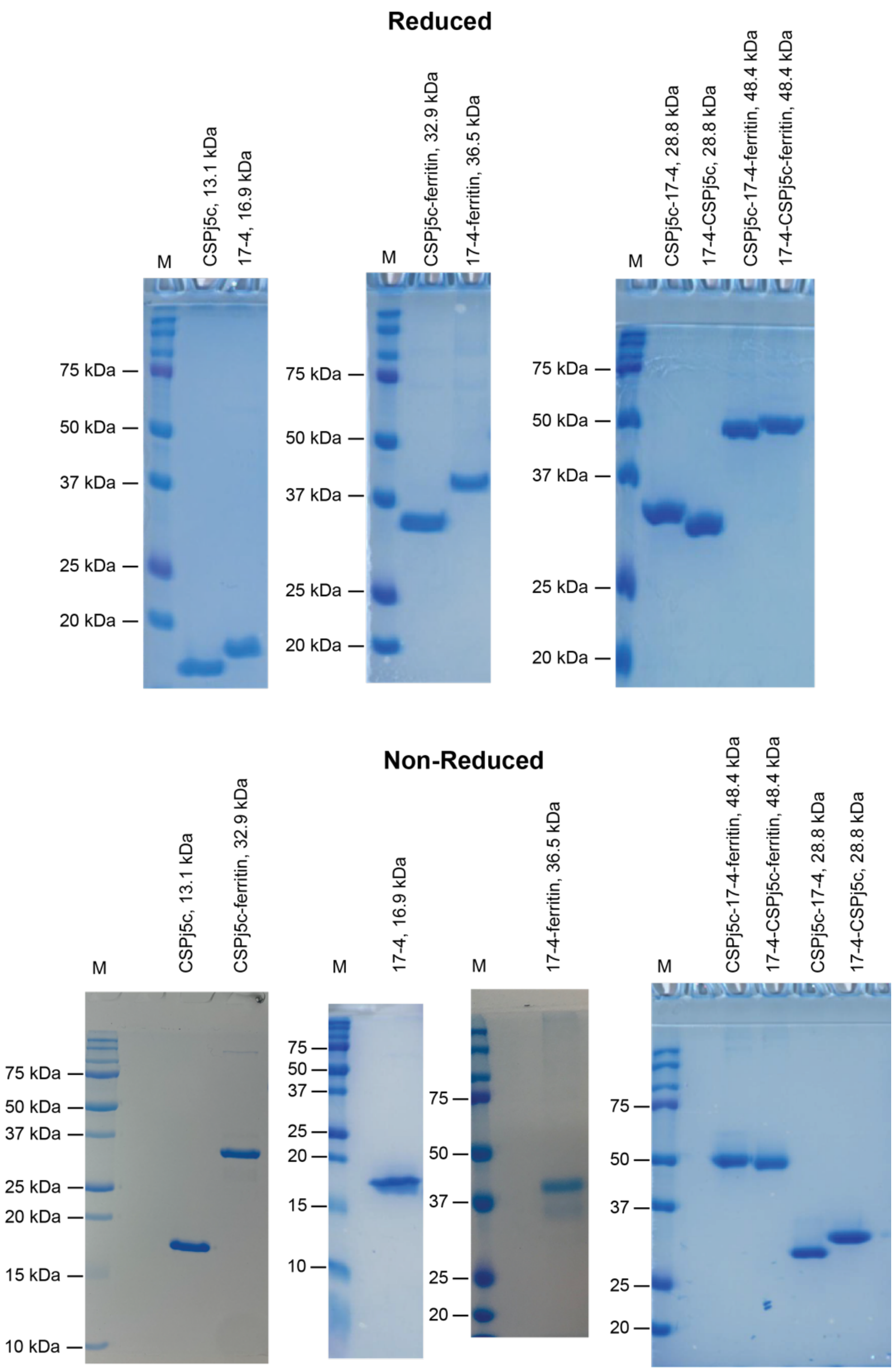

Supplementary Figure 2. Purified proteins on SDS-PAGE. Approximately 2 μg of purified protein was separated on a 12% or 15% Tris-Glycine polyacrylamide gels under reducing or non-reducing conditions and were visualized with coomassie staining. Precision Plus protein marker (M) is used as standard and the sizes (kDa) of the molecular mass markers are indicated.

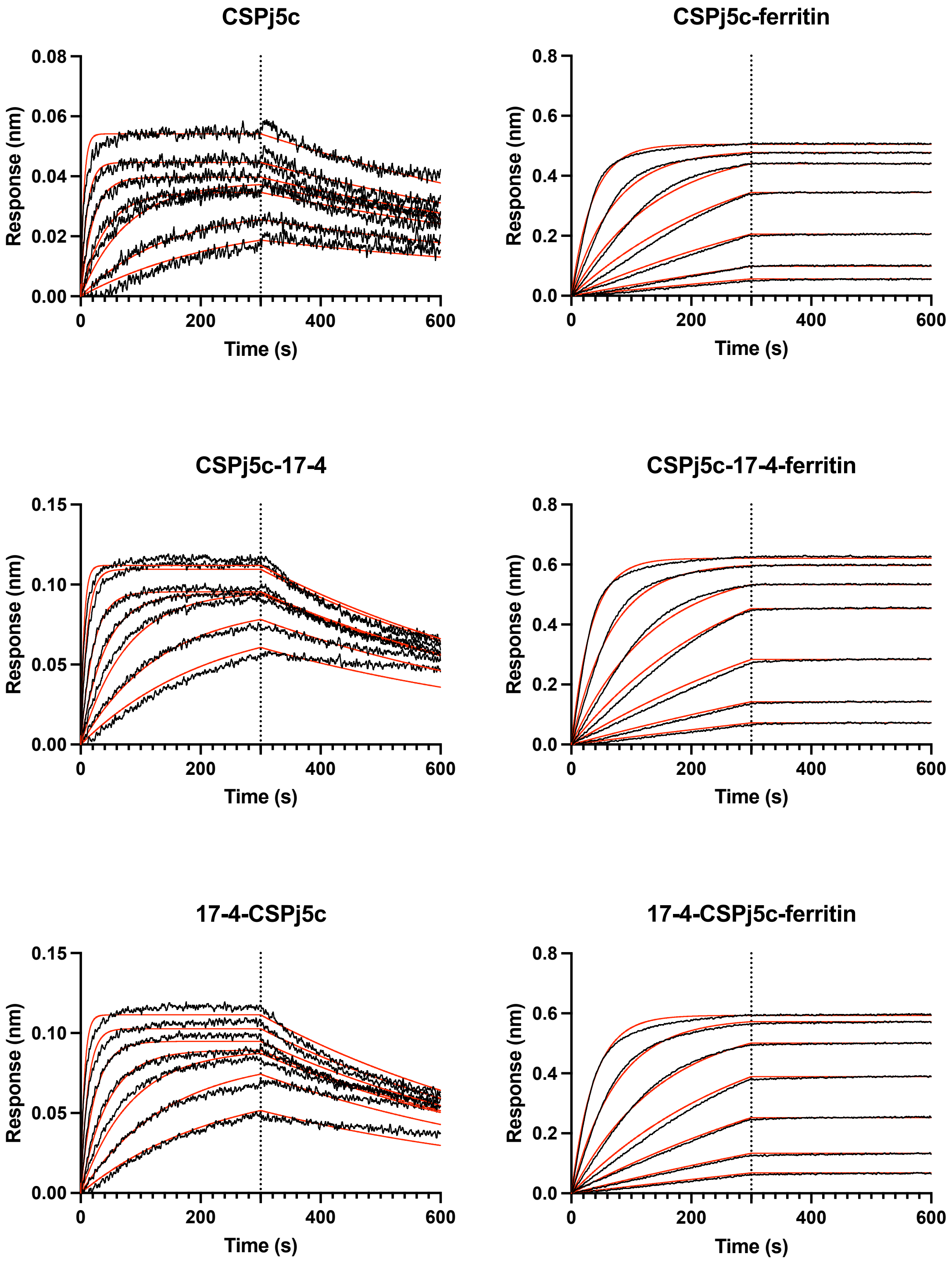

Supplementary Figure 3. Representative BLI traces of CIS43 mAb binding to antigen designs. Two-fold dilution starting from 125 nM of antigen. Raw sensorgrams and fitting curves are shown in black and red, respectively. Association and dissociation phases are divided by the dotted lines.

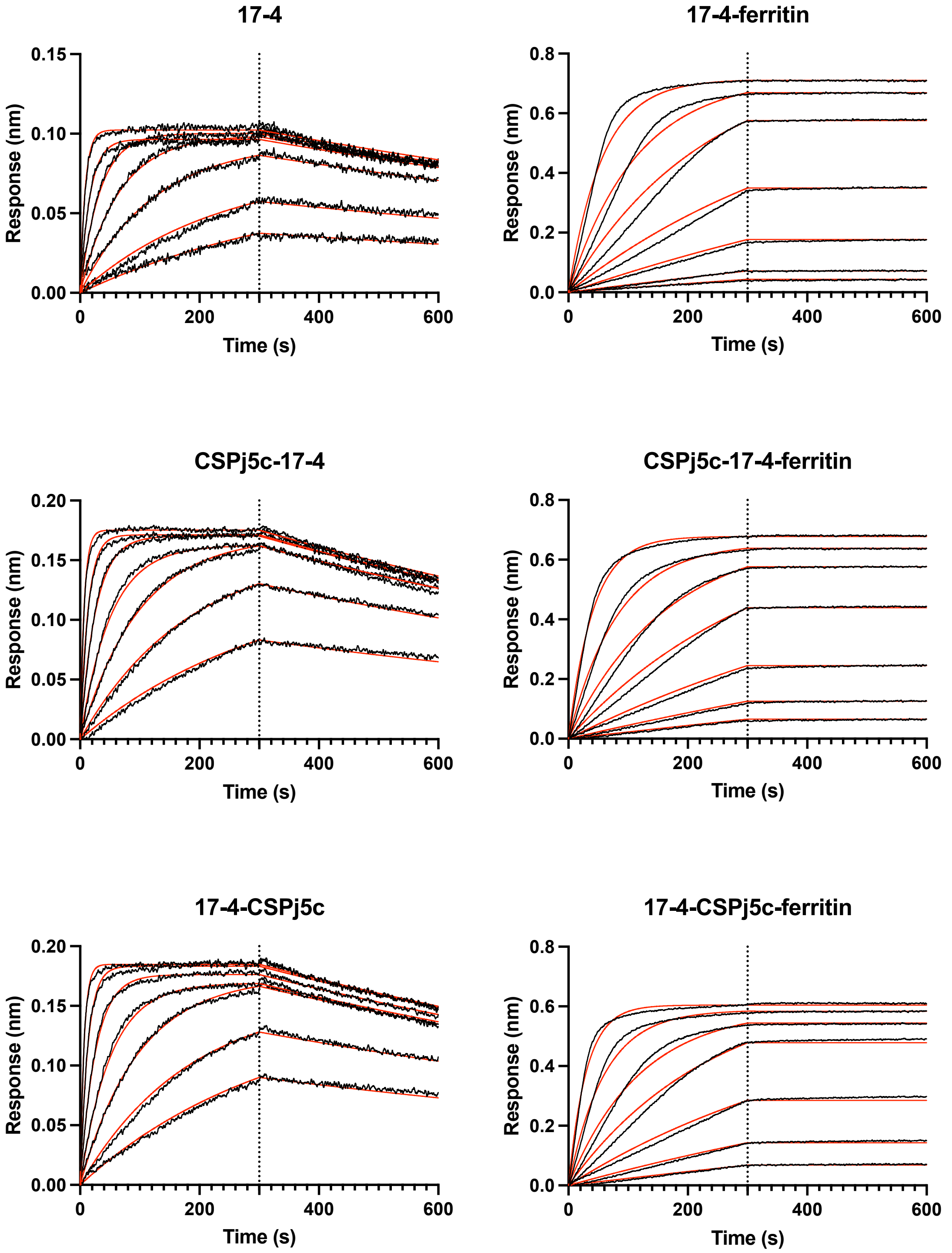

Supplementary Figure 4. Representative BLI traces of TB31F mAb binding to antigen designs. Two-fold dilution starting from 125 nM of antigen. Raw sensorgrams and fitting curves are shown in black and red, respectively. Association and dissociation phases are divided by the dotted lines.
C57BL

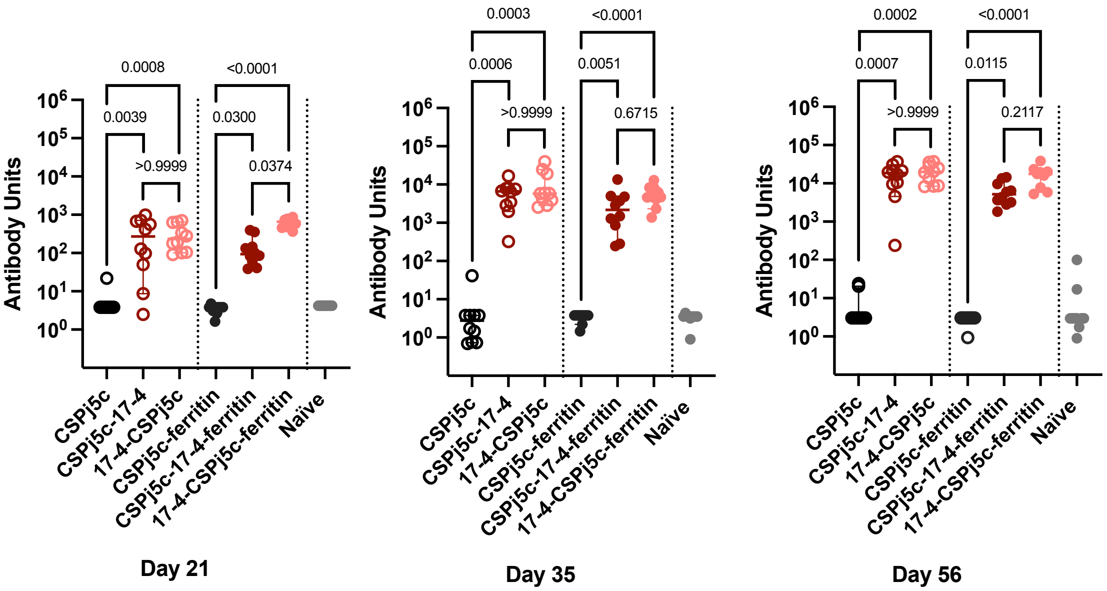

Supplementary Figure 5. Antibody IgG titer against Pfs48/45 WT D3 in C57BL/6 immunized mice at days 21, 35 and 56. Median values and 95% confidence levels are displayed with individual values, and the p-values were calculated using the Kruskal-Wallis non-parametric test followed by Dunn's test corrected for the three comparisons in each section of the graph

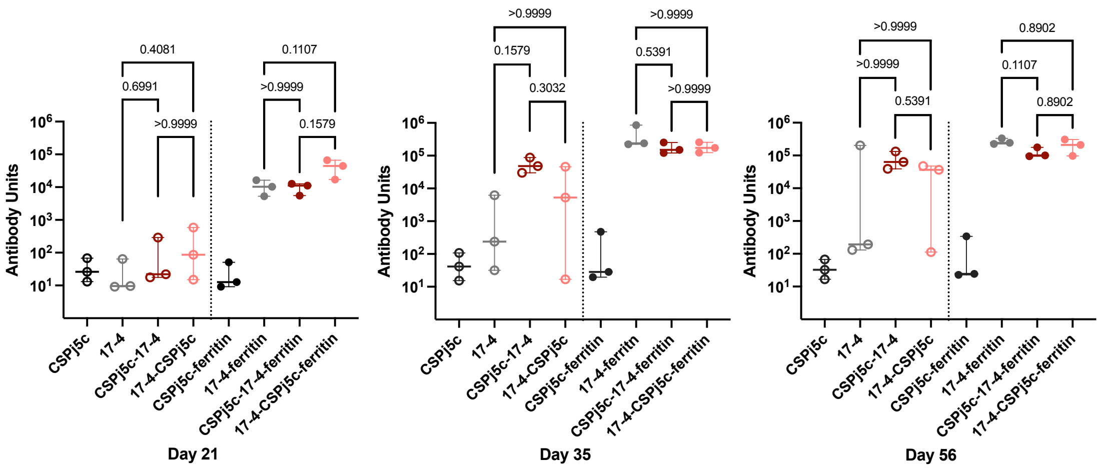

Supplementary Figure 6. Antibody IgG titer against Pfs48/45 WT D3 in immunized rabbits at days 21, 35 and 56. Median and 95% CI values are displayed. The p-values were obtained using Kruskal-Wallis non-parametric test followed by Dunn's multiple comparisons.

Supplementary Table 1. Sequences for antigen designs.

Pfs48/45 17-4	ETGEKKVIHGCNFASNVASKHTFTKSLDISLVDDSAHISCNVHLSEKKYNHLVG MNCPGDIIPDCFFQVYQQESEELEPSNIVYLDSQINIGDIEYYEDAEGDDKIKLFKI VGSIPKTTSFTCICKKDKKSAYMTVTIDSAGGTKHHHHHH
CSPj5c	ETGNPDPNANPNVDPNANPNANPNANPNANPNANPNAEPSDKHIKEYLNKIQNS LSTEWSPCSVTCGNGIQVRIKPGSANKPKDELDYANDIEKKICKMEKCSSVFNVV NSGTKHHHHHH
17-4-CSPj5c	ETGEKKVIHGCNFASNVASKHTFTKSLDISLVDDSAHISCNVHLSEKKYNHLVG MNCPGDIIPDCFFQVYQQESEELEPSNIVYLDSQINIGDIEYYEDAEGDDKIKLFKI VGSIPKTTSFTCICKKDKKSAYMTVTIDSAGNPDPNANPNVDPNANPNANPNANP NANPNANPNAEPSDKHIKEYLNKIQNSLSTEWSPCSVTCGNGIQVRIKPGSANKP KDELDYANDIEKKICKMEKCSSVFNVVNSGGSGTKHHHHHH
CSPj5c-17-4	ETGNPDPNANPNVDPNANPNANPNANPNANPNANPNAEPSDKHIKEYLNKIQNS LSTEWSPCSVTCGNGIQVRIKPGSANKPKDELDYANDIEKKICKMEKCSSVFNVV NSGGSEKKVIHGCNFASNVASKHTFTKSLDISLVDDSAHISCNVHLSEKKYNHLV GMNCPGDIIPDCFFQVYQQESEELEPSNIVYLDSQINIGDIEYYEDAEGDDKIKLFK IVGSIPKTTSFTCICKKDKKSAYMTVTIDSAGGTKHHHHHH
17-4-ferritin	ETGEKKVIHGCNFASNVASKHTFTKSLDISLVDDSAHISCNVHLSEKKYNHLVG MNCPGDIIPDCFFQVYQQESEELEPSNIVYLDSQINIGDIEYYEDAEGDDKIKLFKI VGSIPKTTSFTCICKKDKKSAYMTVTIDSAGGTGSGGGGESQVRQQFSKDIEKLL NEQVNKEMQSSNLYMSMSSWCYTHSLDGAGLFLFDHAAEEYEHAKKLIIFLNE NNVPVQLTSISAPEHKFEGLTQIFQKAYEHEQHISESINNIVDHAIKSKDHATFNFL QWYVAEQHEEEVLFKDILDKIELIGNENHGLYLADQYVKGIAKSRKSGS
CSPj5c-ferritin	ETGNPDPNANPNVDPNANPNANPNANPNANPNANPNAEPSDKHIKEYLNKIQNS LSTEWSPCSVTCGNGIQVRIKPGSANKPKDELDYANDIEKKICKMEKCSSVFNVV NSGGSGTGSGGGGESQVRQQFSKDIEKLLNEQVNKEMQSSNLYMSMSSWCYTH SLDGAGLFLFDHAAEEYEHAKKLIIFLNENNVPVQLTSISAPEHKFEGLTQIFQKA YEHEQHISESINNIVDHAIKSKDHATFNFLQWYVAEQHEEEVLFKDILDKIELIGN ENHGLYLADQYVKGIAKSRKSGS
17-4-CSPj5c-ferritin	ETGEKKVIHGCNFASNVASKHTFTKSLDISLVDDSAHISCNVHLSEKKYNHLVG MNCPGDIIPDCFFQVYQQESEELEPSNIVYLDSQINIGDIEYYEDAEGDDKIKLFKI VGSIPKTTSFTCICKKDKKSAYMTVTIDSAGNPDPNANPNVDPNANPNANPNANP NANPNANPNAEPSDKHIKEYLNKIQNSLSTEWSPCSVTCGNGIQVRIKPGSANKP KDELDYANDIEKKICKMEKCSSVFNVVNSGGSGTGSGGGGESQVRQQFSKDIEK LLNEQVNKEMQSSNLYMSMSSWCYTHSLDGAGLFLFDHAAEEYEHAKKLIIFLN ENNVPVQLTSISAPEHKFEGLTQIFQKAYEHEQHISESINNIVDHAIKSKDHATFNF LQWYVAEQHEEEVLFKDILDKIELIGNENHGLYLADQYVKGIAKSRKSGS
CSPj5c-17-4-ferritin	ETGNPDPNANPNVDPNANPNANPNANPNANPNANPNAEPSDKHIKEYLNKIQNS LSTEWSPCSVTCGNGIQVRIKPGSANKPKDELDYANDIEKKICKMEKCSSVFNVV NSGGSEKKVIHGCNFASNVASKHTFTKSLDISLVDDSAHISCNVHLSEKKYNHLV GMNCPGDIIPDCFFQVYQQESEELEPSNIVYLDSQINIGDIEYYEDAEGDDKIKLFK IVGSIPKTTSFTCICKKDKKSAYMTVTIDSAGGTGSGGGGESQVRQQFSKDIEKLL NEQVNKEMQSSNLYMSMSSWCYTHSLDGAGLFLFDHAAEEYEHAKKLIIFLNE NNVPVQLTSISAPEHKFEGLTQIFQKAYEHEQHISESINNIVDHAIKSKDHATFNFL QWYVAEQHEEEVLFKDILDKIELIGNENHGLYLADQYVKGIAKSRKSGS

Supplementary Table 2. Median titers in C57BL/6 mice over time. p-values relative to naïve control are displayed in parentheses and were calculated by Kruskal-Wallis followed by Dunn's test corrected for 6 comparisons to the naïve group

Sample Name	CSPj5c Titers			17-4 Titers		
	**Day 21**	**Day 35**	**Day 56**	**Day 21**	**Day 35**	**Day 56**
**CSPj5c**	2.700 (>0.9999)	3.883 (>0.9999)	2.700 (>0.9999)	4.244 (>0.9999)	1.983 (>0.9999)	3.283 (>0.9999)
**CSPj5c-17-4**	1937 (0.0004)	97417 (<0.0001)	87667 (<0.0001)	2339 (0.0359)	60533 (0.0004)	113883 (0.0095)
**17-4-CSPj5c**	3195 (0.0002)	86000 (<0.0001)	130400 (<0.0001)	1766 (0.0237)	61250 (<0.0001)	141350 (0.0007)
**CSPj5c-ferritin**	49.47 (0.1370)	58.67 (0.2055)	4693 (0.2887)	4.244 (>0.9999)	1.233 (>0.9999)	3.444 (>0.9999)
**CSPj5c-17-4-ferritin**	7912 (<0.0001)	42467 (<0.0001)	91583 (<0.0001)	2103 (0.0589)	15917 (0.0366)	52067 (0.2703)
**17-4-CSPj5c-ferritin**	7462 (<0.0001)	46167 (<0.0001)	41333 (0.009)	5140 (0.0002)	38550 (0.0026)	70667 (0.0410)

Sample Name	Pfs48/45 WT Titers			Ferritin Titers		
	**Day 21**	**Day 35**	**Day 56**	**Day 21**	**Day 35**	**Day 56**
**CSPj5c**	3.833 (>0.9999)	2.767 (>0.9999)	3.067 (>0.9999)	3.417	1.911	3.300
**CSPj5c-17-4**	271.8 (0.0985)	6627 (0.0001)	18522 (<0.0001)	1.461	2.482	6.583
**17-4-CSPj5c**	234.5 (0.0269)	5815 (<0.0001)	19833 (<0.0001)	1.550	2.983	6.583
**CSPj5c-ferritin**	3.833 (0.8129)	3.800 (>0.9999)	3.067 (>0.9999)	3.100 (>0.9999)	2.822 (>0.9999)	48.67 (0.2474)
**CSPj5c-17-4-ferritin**	93.83 (0.3608)	2183 (0.0084)	5183 (0.0055)	28 (0.0152)	2517 (0.0030)	18312 (<0.0001)
**17-4-CSPj5c-ferritin**	658.3 (0.0003)	4706 (0.0001)	17770 (<0.0001)	135.2 (0.0001)	8445 (<0.0001)	33100 (<0.0001)

Supplementary Table 3. Geometric mean titers in C57BL/6 mice over time.

Sample Name	CSPj5c Titers			17-4 Titers		
	Day 21	Day 35	Day 56	Day 21	Day 35	Day 56
**CSPj5c**	2.015	3.889	8.092	5.052	2.526	4.427
**CSPj5c-17-4**	1086	74673	96130	897.5	40080	86763
**17-4-CSPj5c**	2511	79824	153934	1973	59702	120005
**CSPj5c-ferritin**	52.03	62.26	3671	4.863	1.638	3.182
**CSPj5c-17-4-ferritin**	7803	34108	62678	1919	18668	49319
**17-4-CSPj5c-ferritin**	7964	42215	41695	5492	33610	72491

Sample Name	Pfs48/45 WT Titers			Ferritin Titers		
	Day 21	Day 35	Day 56	Day 21	Day 35	Day 56
**CSPj5c**	4.563	2.475	4.559	3.460	2.012	3.792
**CSPj5c-17-4**	130.9	4317	10677	1.926	2.836	4.050
**17-4-CSPj5c**	248.5	7330	17676	1.638	3.232	5.952
**CSPj5c-ferritin**	3.394	3.217	2.687	2.504	2.812	118.6
**CSPj5c-17-4-ferritin**	106.5	1819	5351	32.07	1887	13127
**17-4-CSPj5c-ferritin**	609.2	4872	14271	124.9	8252	25309

Supplementary Table 4. Kaplan-Maier analysis and Log-rank (Mantel-Cox) test p-values for comparison with naïve mice post-challenge with PbPfCSP SPZ. The p-values in red indicate significance below Bonferroni corrected threshold (0.05/6 = 0.0083).

Sample Name	Log-rank (Mantel-Cox) test p-values
CSPj5c	0.6232
CSPj5c-17-4	0.0052
17-4-CSPj5c	0.0131
CSPj5c-ferritin	0.0849
CSPj5c-17-4-ferritin	0.0025
17-4-CSPj5c-ferritin	0.0014

Supplementary Table 5. Median titers in rabbits over time. p-values relative to a negative control (17-4 for CSPj5c and ferritin titers and CSPj5c for 48/45 titers) are displayed in parentheses and were calculated by Kruskal-Wallis followed by Dunn's test corrected for 6 comparisons to the negative group

Sample Name	CSPj5c Titers			17-4 Titers		
	**Day 21**	**Day 35**	**Day 56**	**Day 21**	**Day 35**	**Day 56**
**CSPj5c**	403.0 (>0.9999)	5013 (>0.9999)	9960 (>0.9999)	50.67	47.10	37.60
**17-4**	36.47	80.33	185.3	14.90 (>0.9999)	263.3 (>0.9999)	1057 (>0.9999)
**CSPj5c-17-4**	1603 (>0.9999)	331867 (0.1517)	299600 (0.6000)	26.63 (>0.9999)	77733 (>0.9999)	110800 (0.6859)
**17-4-CSPj5c**	1027 (>0.9999)	47160 (>0.9999)	98000 (>0.9999)	30.13 (>0.9999)	18467 (>0.9999)	70667 (>0.9999)
**CSPj5c-ferritin**	47733 (0.1517)	349333 (0.1517)	499600 (0.0617)	19.47	43.80	49.93
**17-4-ferritin**	116.1	154.3	168.0	40800 (0.3926)	848533 (0.0095)	824800 (0.0029)
**CSPj5c-17-4-ferritin**	407167 (0.0037)	1497867 (0.0048)	1361067 (0.0095)	21033 (>0.9999)	403067 (0.0895)	259333 (0.1071)
**17-4-CSPj5c-ferritin**	136633 (0.0280)	251600 (0.1517)	430400 (0.1517)	118850 (0.1071)	360067 (0.0745)	290800 (0.0419)

Sample Name	Pfs48/45 WT Titers			Ferritin Titers		
	**Day 21**	**Day 35**	**Day 56**	**Day 21**	**Day 35**	**Day 56**
**CSPj5c**	26.17	41.57	32.37	101.4	132.9	105.8
**17-4**	9.567 (>0.9999)	238.3 (>0.9999)	193.3 (>0.9999)	57.63	79.33	103.6
**CSPj5c-17-4**	22.10 (>0.9999)	47867 (0.8866)	63733 (0.5228)	121.0	252.8	218.3
**17-4-CSPj5c**	86.67 (>0.9999)	5287 (>0.9999)	36683 (>0.9999)	103.1	122.1	99.90
**CSPj5c-ferritin**	12.73	28.20	24.50	12.73 (>0.9999)	207467 (>0.9999)	740267 (0.0705)
**17-4-ferritin**	10297 (0.4539)	239033 (0.0148)	244567 (0.0037)	22597 (0.0423)	540533 (0.0548)	332000 (0.2209)
**CSPj5c-17-4-ferritin**	11160 (0.3926)	149833 (0.0895)	100767 (0.1795)	8280 (0.3313)	483600 (0.1138)	491600 (0.2209)
**17-4-CSPj5c-ferritin**	44487 (0.0419)	171800 (0.0510)	209867 (0.0280)	18183 (0.0186)	792267 (0.0423)	694533 (0.1784)

Supplementary Table 6. Geometric mean titers in rabbits over time.

Sample Name	CSPj5c Titers			17-4 Titers		
	**Day 21**	**Day 35**	**Day 56**	**Day 21**	**Day 35**	**Day 56**
**CSPj5c**	500.2	2775	12988	40.92	44.08	40.72
**17-4**	36.13	108.4	768.4	20.31	407.4	4084
**CSPj5c-17-4**	1023	304059	249143	50.41	94047	120638
**17-4-CSPj5c**	794.0	10488	32526	59.04	3803	11995
**CSPj5c-ferritin**	52937	227227	621551	16.35	45.70	51.05
**17-4-ferritin**	63.22	281.1	214.0	46427	916907	756784
**CSPj5c-17-4-ferritin**	312412	1150152	1058939	24241	337394	221921
**17-4-CSPj5c-ferritin**	126661	344223	416629	113867	456662	344505

Sample Name	Pfs48/45 WT Titers			Ferritin Titers		
	**Day 21**	**Day 35**	**Day 56**	**Day 21**	**Day 35**	**Day 56**
**CSPj5c**	28.63	41.11	33.02	83.96	108.0	85.58
**17-4**	17.82	360.3	1726	79.38	93.64	87.22
**CSPj5c-17-4**	48.48	50111	69193	131.0	199.4	223.6
**17-4-CSPj5c**	91.58	1602	5817	88.59	186.9	115.0
**CSPj5c-ferritin**	18.13	63.93	57.50	2849	125557	634225
**17-4-ferritin**	9598	357318	263610	18942	513861	463445
**CSPj5c-17-4-ferritin**	9198	166288	118796	8973	476356	508248
**17-4-CSPj5c-ferritin**	36956	175831	184592	24184	467840	472339

Supplementary Table 7. Transmission reducing activity and the 95% confidence interval values.

	Sample Name	IgG conc [μg/mL]	% Inhibition			
			Estimate	95% CI Low	95% CI High	p-value
SMFA #392-2	CSPj5c	1,000	-74.4	-267.0	21.8	0.173
		333	-51.1	-230.8	32.9	0.322
	17-4	1,000	63.6	18.6	86.1	0.013
		333	14.1	-97.6	63.1	0.722
	CSPj5c-17-4	1,000	53.0	-8.9	80.9	0.070
		333	-22.7	-160.4	47.2	0.636
	17-4-CSPj5c	1,000	12.8	-93.0	61.3	0.722
		333	35.5	-39.3	71.2	0.266
	CSPj5c-ferritin	1,000	40.6	-25.8	72.4	0.172
		333	7.7	-104.1	59.9	0.846
	17-4-ferritin	1,000	100.0	98.3	100.0	0.001
		333	100.0	99.0	100.0	0.001
	CSPj5c-17-4-ferritin	1,000	100.0	99.3	100.0	0.001
		333	97.4	94.1	99.1	0.001
	17-4-CSPj5c-ferritin	1,000	100.0	99.3	100.0	0.001
		333	89.5	75.3	96.9	0.001
SMFA #386-2	CSPj5c	1,000	19.7	-83.1	65.8	0.630
	17-4	1,000	93.2	81.4	98.8	0.001
	CSPj5c-17-4	1,000	98.3	94.8	99.7	0.001
	17-4-CSPj5c	1,000	41.9	-60.0	83.6	0.253
	CSPj5c-ferritin	1,000	45.3	-30.9	80.1	0.158
	17-4-ferritin	1,000	100.0	96.1	100.0	0.001
	CSPj5c-17-4-ferritin	1,000	100.0	93.5	100.0	0.001
	17-4-CSPj5c-ferritin	1,000	100.0	96.9	100.0	0.001
SMFA #420	CSPj5c-ferritin	333	15.4	-73.5	61.2	0.639
	17-4-ferritin	333	100.0	98.8	100.0	0.0001
		111	74.1	43.3	88.4	0.002
		37	46.3	-16.6	76.3	0.102
	CSPj5c-17-4-ferritin	333	87.0	69.4	94.9	0.001
		111	40.1	-29.9	73.4	0.215
		37	20.4	-76.8	65.4	0.565
	17-4-CSPj5c-ferritin	333	92.6	82.3	97.9	0.001
		111	75.3	39.8	91.3	0.004
		37	50.6	-16.3	80.6	0.094

Supplementary Table 8. Significance values for comparisons between monomer and the ferritin nanoparticle at each concentration of IgG. The statistical significance values were evaluated by the zero-inflated negative binomial (ZINB) model, and Bonferroni corrected p-values are shown. N/A represents comparisons where statistical analysis was not performed. The statistical analysis was conducted using data from two independent feeds at 1,000 μg/mL and one feed (SMFA#392-2) at 333 μg/mL.

Sample Name	IgG conc. [μg/mL]	CSPj5c-ferritin	17-4-ferritin	CSPj5c-17-4-ferritin	17-4-CSPj5c-ferritin
CSPj5c	333	0.2329	N/A	N/A	N/A
	1,000	0.0260	N/A	N/A	N/A
17-4	333	N/A	0.0010	N/A	N/A
	1,000	N/A	0.0010	N/A	N/A
CSPj5c-17-4	333	N/A	N/A	0.0010	N/A
	1,000	N/A	N/A	0.0010	N/A
17-4-CSPj5c	333	N/A	N/A	N/A	0.0010
	1,000	N/A	N/A	N/A	0.0010

Supplementary Table 9. Significance values for comparisons within monomers and within ferritin nanoparticles at each concentration of IgG. The statistical significance values were evaluated by the zero-inflated negative binomial (ZINB) model, and Bonferroni corrected p-values are shown. N/A represents comparisons where statistical analysis was not performed. The statistical analysis was conducted using data from two independent feeds at 1,000 μg/mL and one feed (SMFA#392-2) at 333 μg/mL.

IgG conc.		333 μg/mL	1000 μg/mL
Sample Name		p-value	p-value
17-4	17-4-CSPj5c	1.000	0.003
17-4	CSPj5c-17-4	1.000	0.546
17-4-CSPj5c	CSPj5c-17-4	0.339	0.003
17-4-ferritin	17-4-CSPj5c-ferritin	0.003	N/A
17-4-ferritin	CSPj5c-17-4-ferritin	0.003	N/A
17-4-CSPj5c-ferritin	CSPj5c-17-4-ferritin	0.006	N/A
